# Anticancer Action and Mechanism of Ergosterol Peroxide from *Paecilomyces cicadae* Fermentation Broth

**DOI:** 10.3390/ijms19123935

**Published:** 2018-12-07

**Authors:** Linfu He, Wenjing Shi, Xiaocui Liu, Xiaohuan Zhao, Zhicai Zhang

**Affiliations:** 1 School of Food and Biological Engineering, Jiangsu University, Zhenjiang 212013, China; helinfu@163.com (L.H.); 2221718049@stmail.ujs.edu.cn (W.S.); 2221718008@stmail.ujs.edu.cn (X.L.); 2 School of Life Science, East China Normal University, Shanghai 200241, China; 51171300035@stu.ecnu.edu.cn

**Keywords:** ergosterol peroxide, renal cell carcinoma, apoptosis, cell cycle, *Paecilomyces cicadae*

## Abstract

*Isaria cicadae*, a medicinal food fungus, is a fruit from *Paecilomyces cicadae*. In this study, we purified ergosterol peroxide (EP) from the fermentation broth of *P. cicadae* and investigated its effects on renal cell carcinoma (RCC) cells, in vitro. EP was purified from *P. cicadae* fermentation broth. The human RCC cell line 786-0 was used to analyze the anticancer mechanism of EP and inhibit its effect on cancer cell proliferation, in vitro. EP with a validated structure showed a yield rate of 20.1 mg/L and a purity of 96%. EP significantly inhibited RCC cell growth and clone formation in vitro. In addition, EP suppressed the migration and invasion, triggered the apoptosis, and modulated the cell cycle of RCC cells, in a dose-dependent manner. It also downregulated β-catenin expression. EP could be routinely produced through *P. cicadae*. It fights RCC cells in vitro through multiple mechanisms, including suppressing cell growth, colonization, migration, and invasion, arresting the cell cycle, attenuating β-catenin pathways, and triggering apoptosis.

## 1. Introduction

Renal cell carcinoma (RCC) originates from the renal epithelium. It accounts for more than 90% of cancers in the kidney and about 3% of all lethal, malignant, tumors [1]. As many as 30% of RCC patients experience local recurrence or distant metastasis, and the current five-year survival rate is unsatisfactory [2]. Despite marked advances in RCC treatment with first-line targeted tyrosine kinase inhibitors (TKIs, e.g., sorafenib or sunitinib), new foods or medicines with superior effects and safety in RCC treatment are still in demand. Natural foods and agriculture are highly valuable resources for drug development, and the utilization of their active products is a promising strategy for fighting cancers [3,4]. Traditional Chinese herbs are a popular treatment against various epidemics, including cancer. To date, many effective anti-cancer formulas have been derived from these herbs, under the guidance of the traditional Chinese medicine (TCM) theory [5,6,7]. The TCM entomogenous *Isaria cicadae* is an edible entomogenous fungus, formed in cicada nymphs infected by *Paecilomyces cicadae,* and has been widely used to prevent and treat various diseases [8,9]. In China, it has been used for medicinal and edible purposes for more than 1000 years. It is frequently recommended for the treatment of lung diseases. Recently, the anti-tumor effects of *I. cicadae* have been increasingly observed, such as the antiproliferative and pro-apoptosis properties in gynecological carcinoma cells [8]. Ergosterol peroxide (EP) is one of the major active ingredients in *I. cicadae* (molecular formula: C_28_H_44_O·C_6_H_12_O_7_). It can be extracted from *I. cicadae* through multiple chromatographic techniques. As a characteristic secondary metabolite, EP shows similar activity to *I. cicadae*, such as activating the immunity and exerting anti-cancer/virus effects [10,11,12]. Recently, emerging evidence has pointed to the anti-tumor activity of EP in colorectal cancer, hepatocellular carcinoma, prostate cancer, myeloma, and leukemia [13,14,15,16], with known mechanisms like β-catenin signaling, apoptosis-promoting pathways, and anti-angiogenic pathways targeting JAK2/STAT3 signals, etc. However, reports about EP obtained from *P. cicadae* fermentation, and its role in renal cancer tumors worldwide, are scant. Moreover, limited protocols are available for routine EP production. Previously, EP could be isolated from trichophyton *Schoenleini*, *Inonotus radiatus*, *Cordyceps cicadae*, *Ganoderma lucidum*, *Sarcodon aspratus*, etc., high-performance liquid chromatography or similar column chromatography protocols [12,13,17,18,19]. In our pilot experiments, we explored a process to acquire EP from *P. cicadae* broth, and the submerged fermentation of *P. cicadae* is a promising method to obtain large amounts of EP. In the present study, we aimed to purify EP from *P. cicadae* fermentation broth, explore its anti-RCC effects on a human RCC cell line, and elucidate the underlying mechanisms, at least partially in vitro. We observed the anti-proliferative, invasion/migration inhibitory, pro-apoptotic, and cycle-regulating effects of EP via several potential targetable pathways. This study may provide evidence for EP application in renal cancer treatment.

## 2. Results

### 2.1. Identification of Ergosterol Peroxide (EP) Products

**Figure 1:**
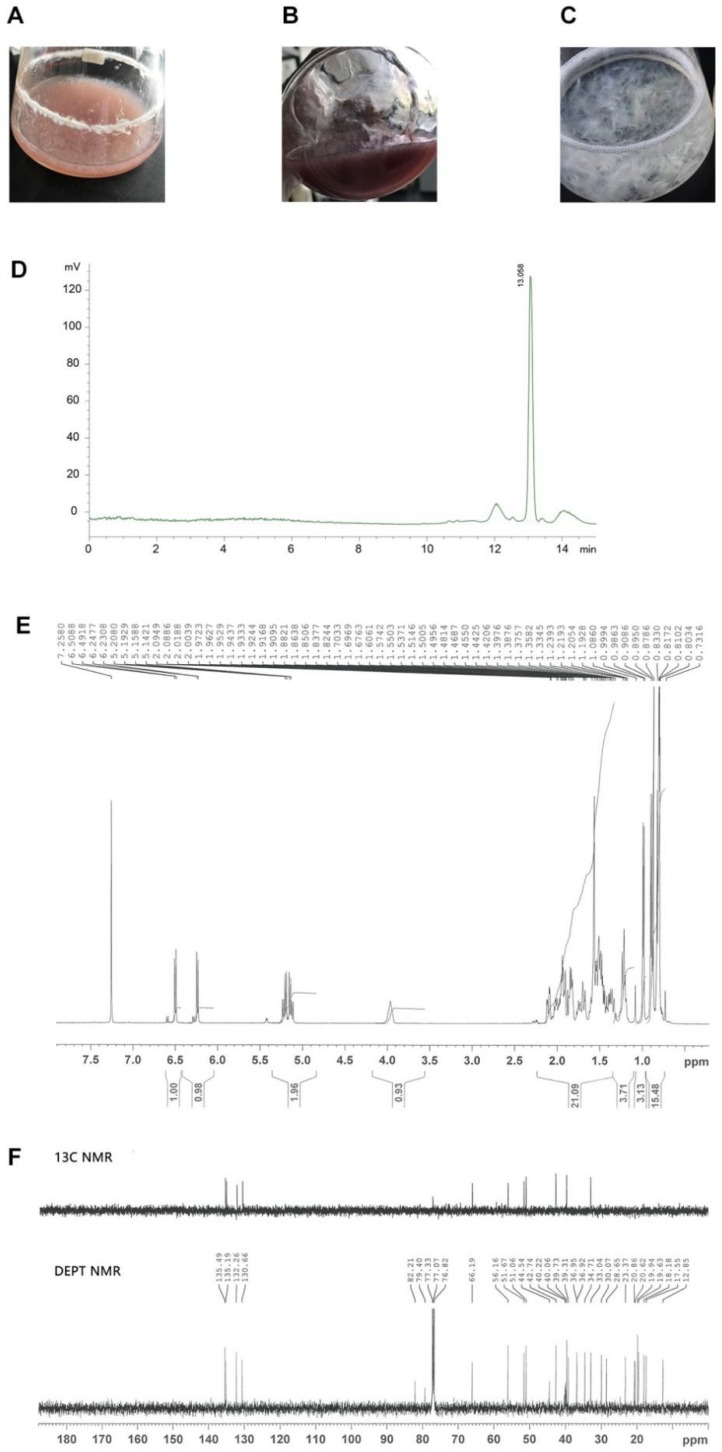
Preparation and identification of ergosterol peroxide (EP). (**A**) *Paecilomyces cicadae* was added and cultured. (**B**) Fermentation products of *P. cicadae*. (**C**) Mycelium of *P. cicadae*. (1**D**) Target fraction and EP had a single peak at 13 min by HPLC. (**E**) Structure of the EP products was determined by ^1^H NMR spectroscopy. (**F**) Identification of EP products by ^13^C and DEPT NMR spectroscopy.

Before anti-cancer studies took place, the following aspects of the EP products were identified. (1) The fraction was dyed by vanillin sulfuric acid and showed a similar color to the positive control, which was purchased form BioBioPha Co., Ltd. (Kunming, China). (2) In HPLC analysis, the target fraction exhibited a single peak at 13 min (Figure 1D). The yield rate was 20.1 mg/L, and the purity reached 96%. The structure of our EP products was determined by: (3) the ^1^H (Figure 1E) and (4) the ^13^C-NMR (Figure 1F) spectroscopy methods. The ^1^H NMR spectrum showed twelve proton signals: 6.59 (^1^H, d, *J* = 8.5 Hz, 7-H), 6.28 (^1^H, d, *J* = 8.5 Hz, 6-H), 5.24 (^1^H, dd, *J* = 7.5, 15.3 Hz, 22-H), 5.16 (^1^H, dd, *J* = 8.0, 15.3 Hz, 23-H), and 4.04 (^1^H, m, 3-H). The ^13^C-NMR (125.77 MHz, CDCl_3_) spectrum calculated a structure of C28H44O3, highly consistent with the positive control and previous reports [20,21].

### 2.2. EP Inhibited In Vitro RCC Cell Growth

**Figure 2:**
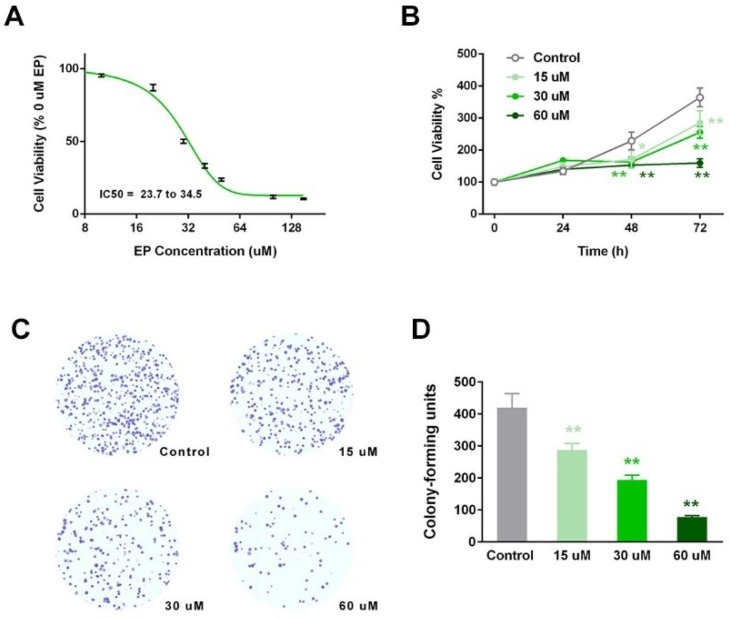
EP inhibited in vitro renal cell carcinoma (RCC)cell growth. (**A**) Suppressive curve of increasing EP concentrations, with an IC50 value of around 30 μM. (**B**) EP groups had a highly significantly lower viability compared with the control. (**C**) Stained colonies in a 12-day soft-agar experiment of four groups. (**D**) A significant decrease in colony number in EP groups. * *p* < 0.05; ** *p* < 0.01, vs. control.

A gradient concentration inhibition curve was drawn, based on cell viability, using the CCK8 method. As shown in Figure 2A, the EP exerted suppressive effects in a dose-dependent manner, with an IC50 value of around 30 μM. Based on this value, we then set three concentrations at 0.5*IC_50_ (15 μM), IC_50_ (30 μM), and 2*IC_50_ (60 μM), in the following observation of the anti-RCC activity of EP. The cell viabilities of the four groups exhibited significant differences after three days of culture (Figure 2B). At 48 h, the 30 and 60 μM groups had a highly significant lower viability compared with the control (*p* < 0.01). At 72 h, all of the EP groups showed highly significant attenuation (*p* < 0.01). Subsequently, we assessed the effect of EP on its in vitro colony forming ability, using the soft agar method. After 12 days of culture, the colonies were stained, and colony units were counted for each group. EP treatment significantly decreased the colony number compared with the control (*p* < 0.01 for three EP groups vs. control), in a dose-dependent manner.

### 2.3. EP Suppressed RCC Cell Migration and Invasion In Vitro

**Figure 3:**
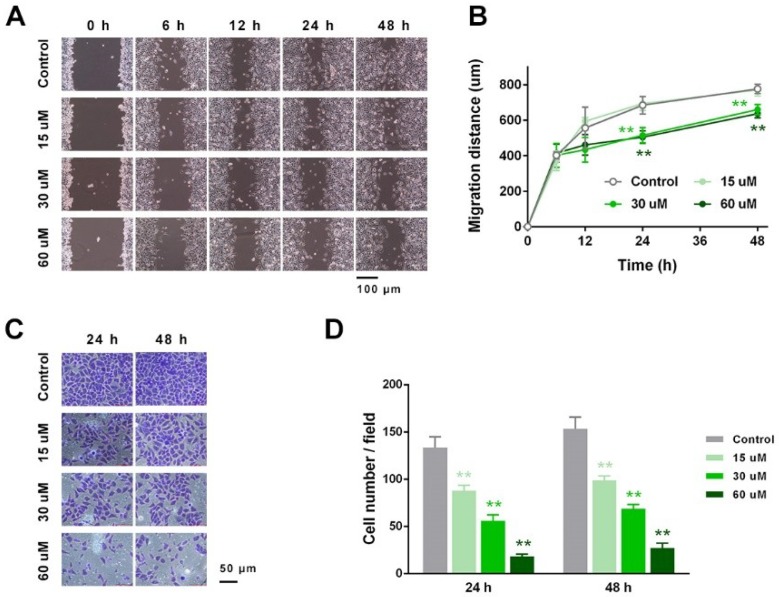
EP suppressed RCC cell migration and invasion. (**A**) Typical photographs of the scratch-assay. (**B**) EP groups displayed much lower migration distance compared with the control group at 24 and 48 h. (**C**) Typical photographs of the invaded cells in the trans-well chamber. (**D**) Number of invaded cells dose-dependently decreased in the EP groups at 24 and 48 h. * *p* < 0.05; ** *p* < 0.01, vs. control.

In vitro migration and invasion were assessed by the scratch-assay and trans-well Matrigel invasion test, respectively. As shown in Figure 3A,B, EP at concentrations above 30 µM exerted a significant suppressive effect. The 30 and 60 µM groups displayed much lower migration distance (larger gaps) than the control group, at 24 and 48 h (*p* < 0.01). Consistently, the trans-well Matrigel experiment showed that EP blocked the RCC cell invasion in a dose-dependent manner, as indicated by the number of invaded cells at 24 and 48 h (at both time points, *p* < 0.05 for 15 μM vs. control, *p* < 0.01 for 30 and 60 μM vs. control) (Figure 3C,D). These results revealed that EP suppressed RCC cell migration/invasion in vitro.

### 2.4. EP Enhanced RCC Cell Apoptosis In Vitro

**Figure 4:**
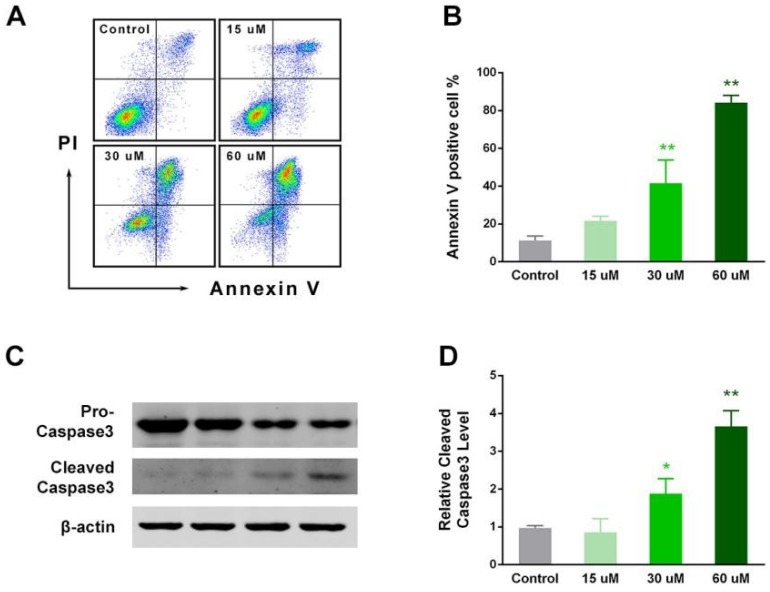
EP enhanced RCC cell apoptosis. (**A**) Typical samples of flow cytometry analysis for Annexin-V and PI-positive cells. (**B**) EP groups had a significantly higher Annexin-V positive ratio compared with the control at 48 h. (**C**) Typical blots of cleaved Caspase-3. (**D**) Cleaved Caspase-3 expression was significantly upregulated in EP groups compared with the control. * *p* < 0.05; ** *p* < 0.01, vs. control.

Apoptosis is a crucial mechanism underlying the anti-cancer effect. The Annexin-V-positive cells were calculated as a level of apoptosis and necrosis, in the flow cytometry analysis. The quadrant was determined from the control. The percentage of Annexin-V-positive cells gradually increased with increasing EP concentration (0, 15, 30, and 60 μM) (F3, 8 = 68.97, *p* < 0.01), and all EP groups had significantly higher apoptosis ratios compared with the control at 48 h (*p* < 0.01 for three EP groups vs. control) (Figure 4A,B). To clarify the direct mechanism, we observed the important apoptotic factor, Caspase-3, using Western blot. The expression of cleaved Caspase-3 was significantly upregulated in the EP groups, compared with the control (*p* < 0.05 for 30 μM vs. control, *p* < 0.01 for 60 μM vs. control) (Figure 4C,D). The above results suggest that EP demonstrates pro-apoptotic activity in in vitro RCC cells.

### 2.5. EP Modulated RCC Cell Cycle in Vitro

**Figure 5:**
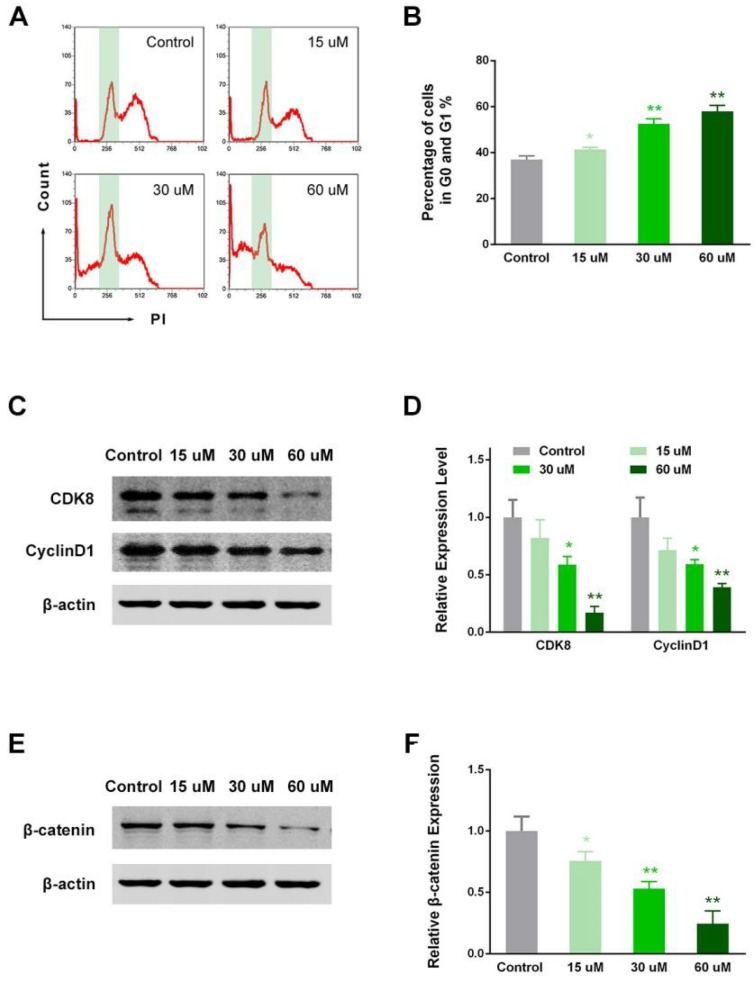
EP modulated RCC cell cycle and suppressed β-catenin expression. (**A**) Typical samples of flow cytometry analysis for cells in different phases, after 48 h of treatment. (**B**) EP groups displayed a dose-dependent increase in G0/G1 cell percentage compared with the control. (**C**) Typical blots of CDK8 and cyclin D1. (**D**) EP groups demonstrated a dose-dependent decrease in CDK8 and cyclin D1 expression. (**E**) Typical blots of β-catenin. (**F**) EP groups showed a dose-dependent decrease in β-catenin. * *p* < 0.05; ** *p* < 0.01 vs. control.

Furthermore, cells in the G0/G1 phase were detected by flow cytometry after Propidium (PI) staining. The percentage of cells in the G0/G1 phase dose-dependently increased after EP treatment (*p* < 0.05 for 15 μM vs. control, *p* < 0.01 for 30 and 60 μM vs. control; Figure 5A,B). This finding suggested that cells in the S–G2 phases decreased, and the EP effectively modulated the RCC cell cycle and blocked DNA replication and mitosis. In support of this, the cycle-related proteins CDK8 and cyclin D1 decreased in expression, particularly in the 30 and 60 μM groups (*p* < 0.01 for 30 and 60 μM vs. control; Figure 5C,D). This finding implied that the cell cycle was another intracellular target regulated by EP. Moreover, we observed β-catenin expression and found that EP exerted dose-dependent inhibition toward β-catenin (Figure 5E,F). Thus, β-catenin-related signaling (e.g., Wnt signaling pathway), may mediate the anti-RCC role of EP.

## 3. Discussion

**Figure 6:**
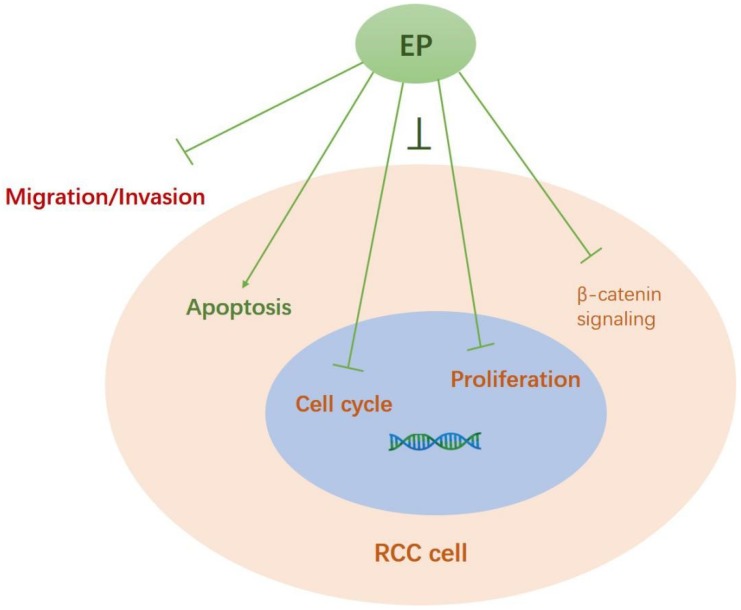
Summarized diagram of the mechanisms underlying EP’s anti-RCC role. EP exerted anti-cancer effects via multiple mechanisms, including inhibiting cell proliferation, attenuating migration/invasion, inducing apoptosis, arresting cell cycles, and suppressing β-catenin signaling.

EP is one of the most common active ingredients in *I. cicadae,* and the most characteristic secondary metabolite of *P. cicadae* [10,11,12]. Our work presented a small-scale protocol to produce EP and proved its anti-RCC activity in vitro. Specifically, EP exerted anti-cancer effects via multiple mechanisms, including inhibiting cell proliferation and clone formation, attenuating migration/invasion, inducing apoptosis, controlling cell cycles, and suppressing β-catenin signaling (as summarized in Figure 6).

This work is the first to focus on the effects of EP on RCC, which clearly highlights the potential application for patients with RCC as one of assistant drug along with current RCC medications.

In the last decade, work has been increasing and many researchers have reported the anti-cancer effects of EP. For example, EP was shown to be cytotoxic against human colorectal cancer cells [22]. Similar to our findings, Wnt/β-catenin signaling may mediate its anti-colorectal cancer and anti-ovarian cancer activities [23,24]. Increasing evidence recently confirmed EP’s anti-tumor activities via apoptosis-promoting pathways or anti-angiogenic pathways, targeting JAK2/STAT3 signals in human hepatocellular carcinoma cells (Hep3B and HepG2), human prostate cancer cells (LNCaP and DU-145 cell lines), and human multiple myeloma U266 cells [13,14,15,25]. These findings were highly consistent with our work. However, until this study, previous work had not yet drawn a conclusion about its role in RCC treatment. Our unpublished work also found that EP may target immune cells and intensify the immune response toward RCC cells and tumors. In addition, EP treatment increased the FOXO3 expression, which may be another mechanism of its anti-RCC role. Thus, EP could target multiple pathways or networks in the anti-cancer process.

In aspects of the in vitro cellular functions that we observed, cell proliferation was consistently inhibited by EP in various cell lines, including ovarian cancer cells, and colon carcinoma cells [24,26]. The anti-migration effect of EP extracted from *Rhizopus oryzae* was also observed in breast cancer cells [27], and the invasion ability of ovarian cancer cells was recently reported [24]. Besides proliferation and migration mechanisms, apoptosis was dramatically enhanced by EP administration, especially in the high dosage group (Figure 4). Caspase-3 is among the key proteases in apoptosis [28]. We observed a significant increase in cleaved caspase-3 expression after EP treatment, indicating that apoptosis may be one of the crucial targets. This phenomenon was also observed in other cancer cells, such as prostate cancer cells [14,29]. Annexin-V-positive cells indicated both apoptosis and necrosis and, overall, EP induced significant cell death in vitro. However, detailed mechanisms were absent in our work. Yang et al. found that EP activates Foxo3-mediated cell death signaling by inhibiting AKT and c-Myc in human hepatocellular carcinoma cells [15]. Their study explained that Foxo3 is an important transcription factor, and its deletion or low expression may cause tumorigenesis and metastasis [30,31]. The cell cycle and apoptosis signaling share some common regulatory networks, and they can act as reciprocal causations. Another study used EP to treat human colon carcinoma HT-29 cells in germinated brown rice, and found a downregulation of cyclin D1, G0/G1 arrest, as well as caspase-3 activation [26]. Combined with our findings, EP simultaneously caused G0/G1 arrest and promoted apoptosis. Shinmoto et al. also used HT-29 cells and reported that EP leads to cell cycle arrest and apoptosis, which might be mediated by CDKN1A [32].

Still, some limitations exist in the present study. Most of the anti-cancer mechanisms in the in vitro experiment had been surveyed previously, in all kinds of cancer studies. Thus, we did not examine any new treatment targets, nor mechanisms of EP’s anti-cancer role. The key significance of this work is that the anti-cancer activity of EP on RCC was investigated for the first time. Besides, we first used the self-purified EP obtained using a fermentation method, instead of a commercial purchase. However, this chemical is already available commercially from Sigma-Aldrich, with more than 97% purity, and our 96% purity had limited significance. Some scholars have used the preparation with 97% purity from Sigma-Aldrich, and investigated its anti-tumor effects on ovarian cancer cells. They also observed an inhibition to the β-catenin pathway, as well as STAT3 [24]. Moreover, we did not observe in vitro angiogenesis, which could be a limitation. Theoretically, angiogenesis plays a contributive role in cancer progress, and directly determines cancer invasion and metastasis. Vascular endothelial growth factor (VEGF) is one of the most widely accepted indicators of the angiogenesis level in tumors or tissues. Previous research claimed a clear inhibition of EP on angiogenesis and VEGF expression [24,25]. In other in vivo work conducted by our team, we found that EP suppresses VEGF in RCC tumor-bearing mice and this may be repeated in cell experiments.

## 4. Materials and Methods

### 4.1. EP Preparation

*P. cicadae* was supplied by the China Microbiological Culture Collection Center (NO. bio-33088). It was inoculated into a 250 mL flask, containing 50 mL of basal medium (20 g/L glucose, 4 g/L yeast extract, 1 g/L peptone, 0.5 g/L K_2_HPO_4_, 0.5 g/L MgSO_4_, pH 5.0; Figure 1A) and cultured for 2.5 days at 26 °C (with 160 rpm shaking), in the dark. The amplified seeds (Figure 1B) were further used for EP generation. Around 5 mL of *P. cicadae* seed was added into 45 mL of PDA medium (200 g/L potato extract, 40 g/L glucose, 4 g/L yeast extract, 1 g/L K_2_HPO_4_, 2 g/L MgSO_4_, pH 5.0) and cultured under identical conditions for 7 days.

Consequently, 50 mL of distilled water was added to dilute the broth, which was then centrifuged at 5000 rpm for 15 min. The precipitate was collected (Figure 1C), washed three times, and dried at 60 °C. Through this protocol, we obtained approximately 73.5 g of dry biomass.

Biomass powder was added into the flask with 500 mL of chloroform/methanol (1:1) solution. The extraction process involved ultrasonic treatment (40 Hz and 250 W) at 25 °C for 1 h, and the solvent was collected. This process was performed three times, and 1500 mL was acquired. The solvent was vacuum distilled at 40 °C and −0.07 MPa for 10 min, and the remaining 200 mg of crude extract was loaded in silica gel chromatography for further purification. After separation, eight fractions were compared with the positive control (EP standard sample). The high-purity fraction was collected, and a second purification was performed using Sephadex LH-20 and a chloroform/methanol (1:1) solution.

The fraction was subjected to vacuum drying. The purified EP was verified using a Bruker DRX 500 MHz superconducting nuclear magnetic resonance spectrometer (Bruker, Karlsruhe, Germany), with CDCI_3_ as solvent, and the ^1^H-NMR, and ^13^C-NMR spectra were analyzed. Validated EP was stored at 4 °C until use.

### 4.2. IC50 Analysis of EP Inhibition on Human RCC Cells

The human RCC cell line 786-0 was supplied by the American Type Culture Collection, and cultured in 10% FBS DMEM, at 37 °C, in a 5% CO_2_ incubator. The inhibitory effects of EP on cell proliferation was evaluated by cell viability assay, following the common CCK8 method. Cells were seeded into 96-well plates (5000 cells/100 µL), and EP was added (with a gradient concentration: 0, 10, 20, 30, 40, 50, 100, and 150 µM) at 6 h after inoculation. The treatment lasted for 48 h before the CCK8 analysis of cell proliferation. Using the absorption at 450 nm, the inhibition curve was drawn and IC_50_ was calculated.

### 4.3. Colony Formation Assay

Colony formation ability was surveyed using the soft agar method. In brief, 768-0 cells (500 cells/well) were mixed with 0.25% low melting agarose gel, containing different concentrations of EP (vehicle, 15, 30, and 60 µM). The mixtures were seeded in six-well plates, which were pre-coated with 0.66% regular agarose gel. The plates were maintained in a CO_2_ incubator for 12 days, and colonies were fixed with 4% formaldehyde for 10 min. Wells were rinsed again with PBS and stained with 0.2% crystal violet solution, in 10% ethanol, for 10 min. Excess stain solution was removed by PBS washing.

### 4.4. Cell Migration Assay

Cell migration was evaluated by using a wound scratch test. Briefly, 3 × 10^5^ 768-0 cells were seeded in 6-well plates. After sufficient adhesion, 4 groups were labelled according to EP concentration, namely, control, 15, 30, and 60 µM. First, the adhered cells were scratched using a pipette tip, and the cultures were washed with PBS to remove debris. Cell migration was monitored daily, and microscope images were captured. The migration distance from the initial scratch site was quantified.

### 4.5. Cell Invasion Assay

For the invasion ability assessment, the trans-well inserts were loaded with 80 μL Matrigel and incubated at 37 °C for 30 min, to allow gel bed formation. The inserts were placed into a 24-well plate containing EP at different concentrations (control, 15, 30, and 60 µM). Afterward, 10^4^ 768-0 cells in 100 μL medium, containing different EP concentrations as above, were gently loaded on top of each gel bed, followed by incubation at 37 °C for 48 h. To observe the trans-welled cells, the inserts were fixed with methanol for 15 min and stained with Coomassie Brilliant Blue. The upper Matrigel layer with cells was cleared, and the cells that invaded through the Matrigel were photographed under a light microscope. Finally, the cells in each field were counted, and four fields were averaged for each well.

### 4.6. Flow Cytometry for Apoptosis and Cell Cycle Analysis

As described above, the four groups were divided, and a 24 h treatment was performed before the assay. Cell apoptosis was analyzed by using an Annexin-V apoptosis detection kit APC (Biolegend, San Diego, CA, USA). In brief, the cultured cells were washed, trypsinized, collected, and resuspended. Afterward, the cells were incubated with Annexin-V in the dark for 15 min, and then washed and resuspended in 400 μL binding buffer, which was finally added with 5 μL propidium iodide solution. The cells were analyzed by FACS Calibur flow cytometry (Biolegend). The percentages of apoptotic and necrotic cells were calculated by using the system. For cell cycle analysis, the cells in the logarithmic phase were harvested. They were then resuspended in cold PBS, and incubated in 70% ethanol, for 3 h. Afterwards, cells were centrifuged at 1500 rpm for 10 min, resuspended in propidium iodide (PI) master mix (40 mg/mL PI and 100 mg/mL RNase in PBS) at a density of 10^5^ cells/mL, and incubated at 37 °C for 30 min, before analysis. After that, flow cytometry analysis was carried out to explore the cell proportion in each phase. The first peak indicating Go/G1 phase, the S phase (the flat region), and the second peak indicating G2 phase were divided; the cell proportion in each phase was automatically calculated by the FACS Calibur flow cytometry system.

### 4.7. Western Blotting

At 48 h after treatment of the four groups, cells were lysed in lysis buffer with protein inhibitor and Phenylmethanesulfonyl fluoride (PMSF, 1 mM). The lysates were centrifuged at 10,000 rpm at 4 °C for 10 min, and supernatants were collected. Afterwards, the protein concentrations were determined; moreover, equal amounts of sample (about 20 μg) were separated using SDS-PAGE, and proteins were transferred onto nitrocellulose membranes. The membranes were incubated in blocking buffer with 5% milk, and then stained with primary antibodies against Pro-Caspase3, Cleaved Caspase3, β-actin, and CDK8, at 4 °C overnight. After washing with Tris Buffered saline Tween (TBST) thrice, the membranes were incubated with goat anti-rabbit or goat anti-mouse hydrogen-peroxide (HRP) conjugated secondary antibodies, at room temperature for 2 h. After washing, membranes were visualized with ECL kits (Merck Millipore, Burlington, MA, USA). The β-actin levels were probed as an endogenous reference.

### 4.8. Statistical Analysis

The in vitro experiments were performed in triplicate, or as specifically indicated. All data were expressed as the mean ± SD. Comparisons among groups were based on one-way ANOVA and post-hoc analyses. *p* < 0.05 was set as statistically significant.

## 5. Conclusions

EP can be routinely produced through *P. cicadae,* using our technology. After being treated with the purified EP production in vitro, the RCC cells were suppressed in terms of growth and colonization, and migration and invasion. Moreover, the treatment arrested the cell cycle, attenuated the β-catenin pathways, and triggered apoptosis in the RCC cells.

## Abbreviations

EP	ergosterol peroxide
RCC	renal cell carcinoma
*P. cicadae*	*Paecilomyces cicadae*
TCM	traditional Chinese medicine
PI	propidium iodide

## References

[1] Cai W., Huang J., Yuan Y., Hu X., Li M., Kong W., Zhang J., Guo J., Chen Y., Huang Y. (2018). Sunitinib or Sorafenib as Neoadjuvant Therapy May not Improve the Survival Outcomes of Renal Cell Carcinoma with Tumor Thrombus. Urol. Int..

[2] Breen D.J., King A.J., Patel N., Lockyer R., Hayes M. (2018). Image-guided Cryoablation for Sporadic Renal Cell Carcinoma: Three- and 5-year Outcomes in 220 Patients with Biopsy-proven Renal Cell Carcinoma. Radiology.

[3] Liu Z.L., Zhu W.R., Zhou W.C., Ying H.F., Zheng L., Guo Y.B., Chen J.X., Shen X.H. (2014). Traditional Chinese medicinal herbs combined with epidermal growth factor receptor tyrosine kinase inhibitor for advanced non-small cell lung cancer: A systematic review and meta-analysis. J. Integr. Med..

[4] Jia L., Ma S., Hou X., Wang X., Qased A.B., Sun X., Liang N., Li H., Yi H., Kong D. (2013). The synergistic effects of traditional Chinese herbs and radiotherapy for cancer treatment. Oncol. Lett..

[5] Zhu H., Hao J., Niu Y., Liu D., Chen D., Wu X. (2018). Molecular targets of Chinese herbs: A clinical study of metastatic colorectal cancer based on network pharmacology. Sci. Rep..

[6] Zhong Z., Qiang W.W., Tan W., Zhang H., Wang S., Wang C., Qiang W., Wang Y. (2016). Chinese Herbs Interfering with Cancer Reprogramming Metabolism. Evid. Based Complement. Altern. Med..

[7] Hong M., Wang N., Tan H.Y., Tsao S.W., Feng Y. (2015). MicroRNAs and Chinese Medicinal Herbs: New Possibilities in Cancer Therapy. Cancers.

[8] Sun Y.F., Sun Y., Wang Z.A., Han R.L., Lu H.F., Zhang J.L., Liu H.T., Wang S.X., Wang P., Dian L.L. (2017). Isaria cicadae conidia possess antiproliferative and inducing apoptosis properties in gynaecological carcinoma cells. Mycology.

[9] Xu Z., Yan X., Song Z., Li W., Zhao W., Ma H., Du J., Li S., Zhang D. (2018). Two heteropolysaccharides from Isaria cicadae Miquel differ in composition and potentially immunomodulatory activity. Int. J. Biol. Macromol..

[10] Matsueda S., Shimoyama M., Imaizumi T., Tsushima Y. (1982). Studies on fungal products. VI. Biological effects of ergosterol-5,8-peroxide. Yakugaku Zasshi J. Pharm. Soc. Jpn..

[11] Lindequist U., Lesnau A., Teuscher E., Pilgrim H. (1989). The antiviral action of ergosterol peroxide. Die Pharm..

[12] Kahlos K., Kangas L., Hiltunen R. (1989). Ergosterol peroxide, an active compound from *Inonotus radiatus*. Planta Med..

[13] Chen Y.K., Kuo Y.H., Chiang B.H., Lo J.M., Sheen L.Y. (2009). Cytotoxic activities of 9,11-dehydroergosterol peroxide and ergosterol peroxide from the fermentation mycelia of *Ganoderma lucidum* cultivated in the medium containing leguminous plants on Hep 3B cells. J. Agric. Food Chem..

[14] Russo A., Cardile V., Piovano M., Caggia S., Espinoza C.L., Garbarino J.A. (2010). Pro-apoptotic activity of ergosterol peroxide and (22*E*)-ergosta-7,22-dien-5α-hydroxy-3,6-dione in human prostate cancer cells. Chem. Biol. Interactions.

[15] Li X., Wu Q., Bu M., Hu L., Du W.W., Jiao C., Pan H., Sdiri M., Wu N., Xie Y. (2016). Ergosterol peroxide activates Foxo3-mediated cell death signaling by inhibiting AKT and c-Myc in human hepatocellular carcinoma cells. Oncotarget.

[16] Kobori M., Yoshida M., Ohnishi-Kameyama M., Shinmoto H. (2007). Ergosterol peroxide from an edible mushroom suppresses inflammatory responses in RAW264.7 macrophages and growth of HT29 colon adenocarcinoma cells. Br. J. Pharmacol..

[17] Nam K.S., Jo Y.S., Kim Y.H., Hyun J.W., Kim H.W. (2001). Cytotoxic activities of acetoxyscirpenediol and ergosterol peroxide from *Paecilomyces tenuipes*. Life Sci..

[18] Bauslaugh G., Just G., Blank F. (1964). Isolation of Ergosterol Peroxide from *Trichophyton Schoenleini*. Nature.

[19] Takei T., Yoshida M., Ohnishi-Kameyama M., Kobori M. (2005). Ergosterol peroxide, an apoptosis-inducing component isolated from *Sarcodon aspratus* (Berk.) S. Ito. Biosci. Biotechnol. Biochem..

[20] Zhu R., Zheng R., Deng Y., Chen Y., Zhang S. (2014). Ergosterol peroxide from Cordyceps cicadae ameliorates TGF-β1-induced activation of kidney fibroblasts. Phytomed. Int. J. Phytother. Phytopharm..

[21] Bok J.W., Lermer L., Chilton J., Klingeman H.G., Towers G.H. (1999). Antitumor sterols from the mycelia of *Cordyceps sinensis*. Phytochemistry.

[22] Lee J.S., Ma C.M., Park D.K., Yoshimi Y., Hatanaka M., Hattori M. (2008). Transformation of ergosterol peroxide to cytotoxic substances by rat intestinal bacteria. Biol. Pharm. Bull..

[23] Kang J.H., Jang J.E., Mishra S.K., Lee H.J., Nho C.W., Shin D., Jin M., Kim M.K., Choi C., Oh S.H. (2015). Ergosterol peroxide from Chaga mushroom (*Inonotus obliquus*) exhibits anti-cancer activity by down-regulation of the β-catenin pathway in colorectal cancer. J. Ethnopharmacol..

[24] Tan W., Pan M., Liu H., Tian H., Ye Q., Liu H. (2017). Ergosterol peroxide inhibits ovarian cancer cell growth through multiple pathways. Oncol. Targets Ther..

[25] Rhee Y.H., Jeong S.J., Lee H.J., Lee H.J., Koh W., Jung J.H., Kim S.H., Sung-Hoon K. (2012). Inhibition of STAT3 signaling and induction of SHP1 mediate antiangiogenic and antitumor activities of ergosterol peroxide in U266 multiple myeloma cells. BMC Cancer.

[26] Park H.J., Choi S.Y., Hong S.M., Hwang S.G., Park D.K. (2010). The ethyl acetate extract of *Phellinus linteus* grown on germinated brown rice induces G0/G1 cell cycle arrest and apoptosis in human colon carcinoma HT29 cells. Phytother. Res..

[27] Lee D.Y., Lee S.J., Kwak H.Y., Jung L., Heo J., Hong S., Kim G.W., Baek N.I. (2009). Sterols isolated from Nuruk (*Rhizopus oryzae* KSD-815) inhibit the migration of cancer cells. J. Microbiol. Biotechnol..

[28] Wen H., Wu Z., Hu H., Wu Y., Yang G., Lu J., Yang G., Guo G., Dong Q. (2018). The anti-tumor effect of pachymic acid on osteosarcoma cells by inducing PTEN and Caspase 3/7-dependent apoptosis. J. Nat. Med..

[29] Zhu Y.T., Li F., Han B., Tighe S., Zhang S., Chen S.Y., Liu X., Tseng S.C. (2014). Activation of RhoA-ROCK-BMP signaling reprograms adult human corneal endothelial cells. J. Cell Biol..

[30] Hagenbuchner J., Rupp M., Salvador C., Meister B., Kiechl-Kohlendorfer U., Muller T., Geiger K., Sergi C., Obexer P., Ausserlechner M.J. (2016). Nuclear FOXO3 predicts adverse clinical outcome and promotes tumor angiogenesis in neuroblastoma. Oncotarget.

[31] Lee J., Park S.H. (2016). Tumor-suppressive activity of 1,25-dihydroxyvitamin D3 against kidney cancer cells via up-regulation of FOXO3. Biosci. Biotechnol. Biochem..

[32] Barker B.M., Kroll K., Vodisch M., Mazurie A., Kniemeyer O., Cramer R.A. (2012). Transcriptomic and proteomic analyses of the *Aspergillus fumigatus* hypoxia response using an oxygen-controlled fermenter. BMC Genom..

